# Supplementary material for: A Multi-Omics Study of Epigenetic Changes in Type II Alveolar Cells of A/J Mice Exposed to Environmental Tobacco Smoke
Source: Int J Mol Sci. 2024 Aug 29;25(17):9365. doi: 10.3390/ijms25179365 (PMC11394788; doi:10.3390/ijms25179365)

Supplementary files for

A Multi-Omics Study of Epigenetic Changes in Type II Alveolar Cells of A/J Mice Exposed to Environmental Tobacco Smoke

Qiyuan Han, Jenna Fernandez, Andrew T. Rajczewski, Thomas J. Y. Kono, Nicholas A. Weirath, Abdur Rahim, Alexander S. Lee, Donna Seabloom and Natalia Y. Tretyakova

Table S1. Body weight of mice with 10 weeks air or ECS exposure.

Gender	Filtered Air Control	ECS
Male	24.68 ± 2.49 g	21.34 ± 1.37 g
Female	19.41± 1.19 g	16.86 ± 0.62 g

Table S2. Lung histopathology results for A/J mice treated with cigarette smoke.

CPSR ID	Investigator Animal ID	Cohort	Microscopic findings	Macrophage score*
20-016-1	10FC1	10 weeks female air control	NSF	0
20-016-2	10FC2	10 weeks female air control	NSF	0
20-016-3	10FC3	10 weeks female air control	NSF	0
20-016-4	10FC4	10 weeks female air control	NSF	0
20-016-5	10FS1	10 weeks female ECS	Widespread focally variable vascular congestion with multifocal alveolar collapse/atelectasis (interpreted as artifact). Multifocally minimally increased alveolar macrophages. Occasional intra-alveolar and rare intra-bronchiolar pigment-laden macrophages.	1
20-016-6	10FS2	10 weeks female ECS	Multifocally minimally increased alveolar macrophages. One lobe with moderate to marked congesiton and marked collapse of alveolar spaces/atelectasis (interpreted as artifact). Rare intra-alveolar and occasional intra-bronchiolar pigment-laden macrophages.	1
20-016-7	10MC1	10 weeks male air control	NSF	0
20-016-8	10MC2	10 weeks male air control	NSF	0
20-016-9	10MC3	10 weeks male air control	NSF	0
20-016-10	10MS1	10 weeks male ECS	Mild multifocal vascular congestion. Rare intra-alveolar and rare intra-bronchiolar pigment-laden macrophages.	1
20-016-11	10MS2	10 weeks male ECS	Focal mild hemorrhage (at cut margin; interpreted as artifact). Separate focus of hemorrhage/congestion with serum protein/coagulation extending beyond border of lung tissue (interpreted as artifact and/or agonal). Occasional intra-bronchiolar and rare intra-alveolar pigment-laden macrophages.<br>Large section of liver tissue included.	1
20-016-12	10MS3	10 weeks male ECS	Rare foci with small amount of proteinaceous material and rare pigment-laden macrophages. Adjacent tissue is compressed (interpreted as artifact), hindering complete interpretation. Widespread mild to moderate vascular congestion. Rare intra-bronchiolar and rare intra-alveolar pigment-laden macrophages.	1
20-016-13	14FC1	Post exposure female air control	NSF	0
20-016-14	14FC2	Post exposure female air control	NSF	0
20-016-15	14FC3	Post exposure female air control	NSF	0
20-016-16	14FS1	Post exposure female ECS	NSF	0
20-016-17	14FS2	Post exposure female ECS	Single focus of few pigment-laden macrophages in alveolar space. Single focus of few pigment-laden macrophages in peribronchiolar tissue.	1

CPSR ID	Investigator Animal ID	Cohort	Microscopic findings	Macrophage score*
20-016-18	14FS3	Post exposure female ECS	Minimally increased alveolar macrophages. Occasional intra-alveolar and rare intra-bronchiolar pigment-laden macrophages.	1
20-016-19	14MC1	Post exposure male air control	Minimally increased alveolar macrophages. Rare intra-bronchiolar and rare intra-alveolar pigment-laden macrophages.	1
20-016-20	14MC2	Post exposure male air control	NSF	0
20-016-21	14MC3	Post exposure male air control	NSF	0
20-016-22	14MC4	Post exposure male air control	NSF	0
20-016-23	14MS1	Post exposure male ECS	Rare intra-bronchiolar and rare intra-alveolar pigment-laden macrophages.	1
20-016-24	14MS2	Post exposure male ECS	Minimally increased alveolar macrophages. Rare intra-bronchiolar and rare intra-alveolar pigment-laden macrophages.	1
20-016-25	14MS3	Post exposure male ECS	Minimally increased alveolar macrophages. Rare intra-bronchiolar and rare intra-alveolar pigment-laden macrophages.	1
20-016-26	10PDM1	Died during smoke exposure period	Multifocal intra-bronchiolar and intra-alveolar pigment-laden macrophages. Mild multifocal intra-aveolar hemorrhage and mild focal intra-alveolar edema.	1
20-016-27	10PDM2	Died during smoke exposure period	Multifocal intra-bronchiolar and intra-alveolar pigment-laden macrophages. Mild multifocal intra-aveolar hemorrhage.	1
20-016-28	10PDF1	Died during smoke exposure period	Multifocal intra-bronchiolar and intra-alveolar pigment-laden macrophages. Minimally increased alveolar macrophages. Mild multifocal intra-aveolar hemorrhage.	1

Figure S1. Plot showing survival analysis

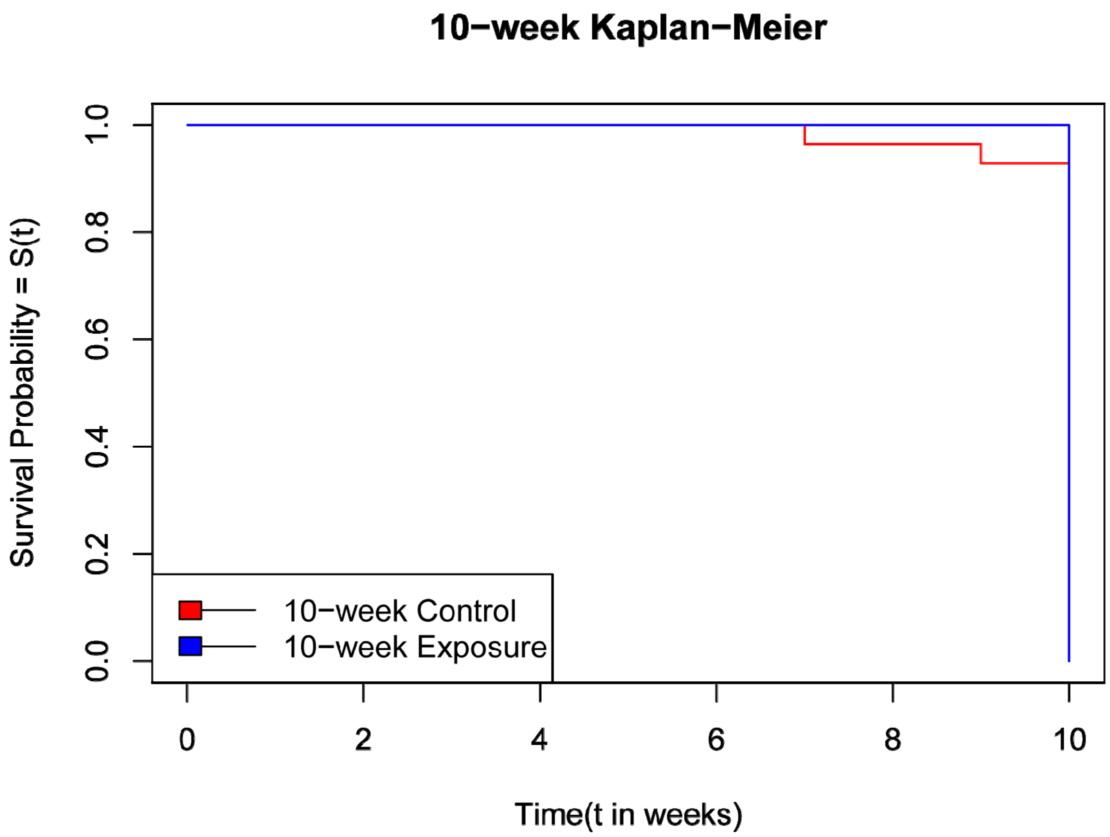

Figure S2. Global genomic levels of 5meC and 5hmC in liver DNA of A/J mice exposed to ECS. Data are expressed as percent of dC and represents mean values ± SD of at least three animals. The specific treatment and duration are shown on the x-axis. *p < 0.05, **p < 0.01, ***p < 0.001.

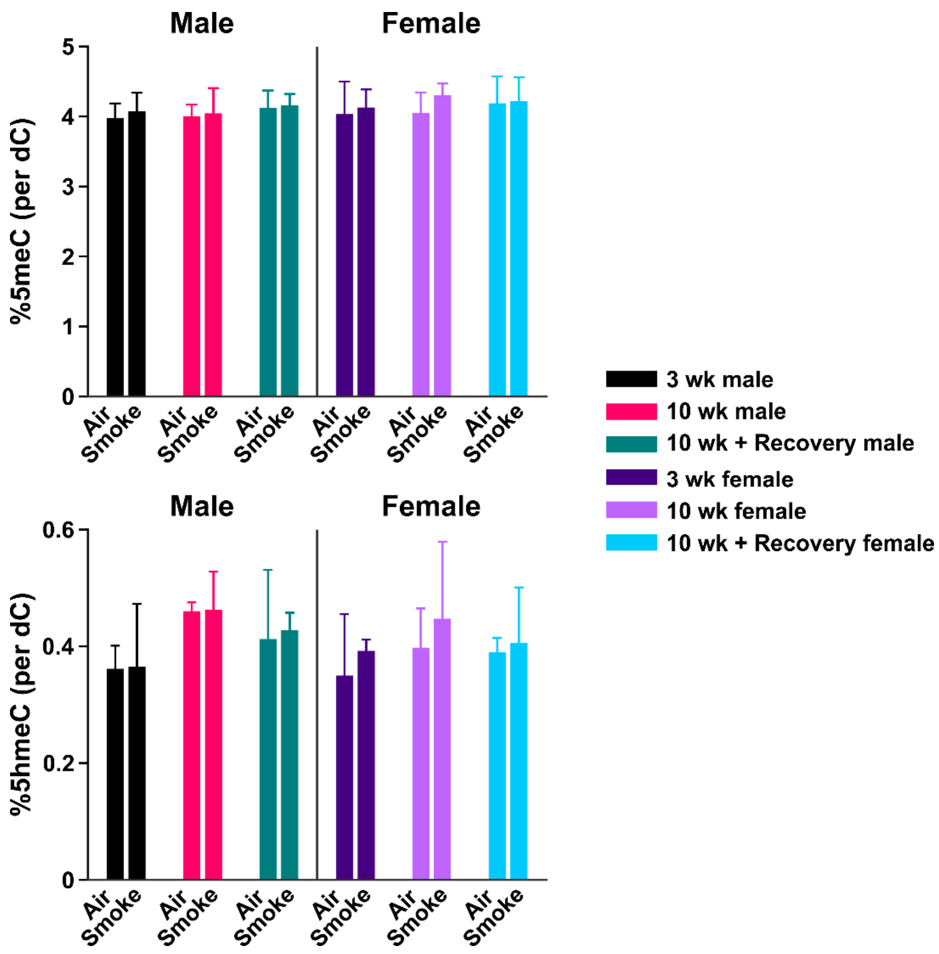

Figure S3. Venn diagram to show the overlap of DhMR among different exposure groups, indicating a reversibility of DhMR.

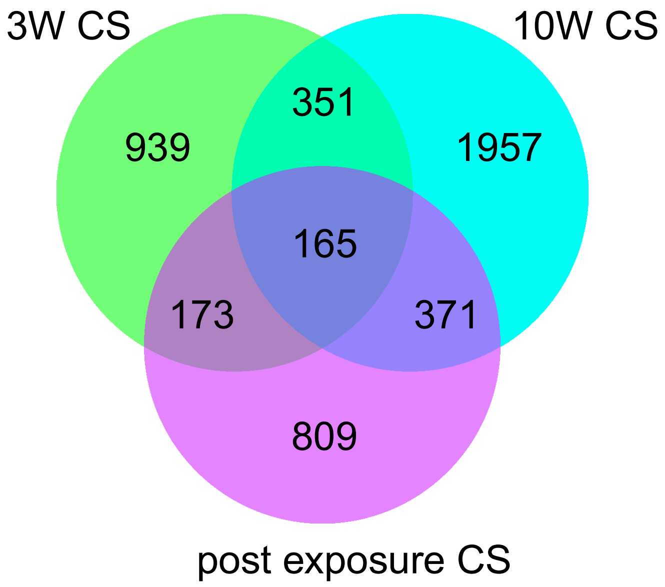

Figure S4. Venn diagram to show the overlap of DEG among different exposure groups, indicating a reversibility of gene expression.

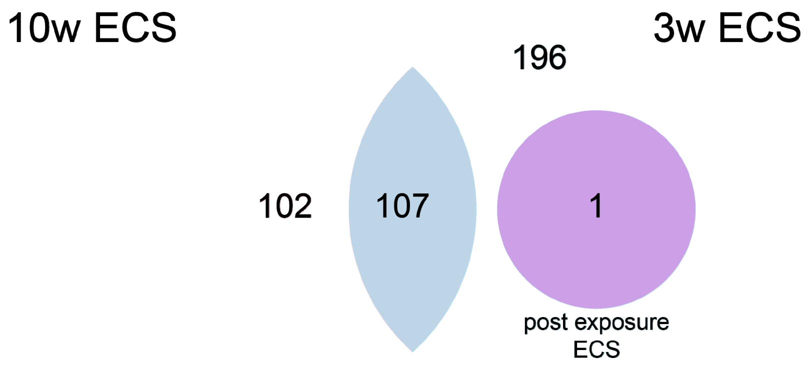

Figure S5. Protein abundance changes in type II pneumocyes of A/J male mice exposed to ECS.

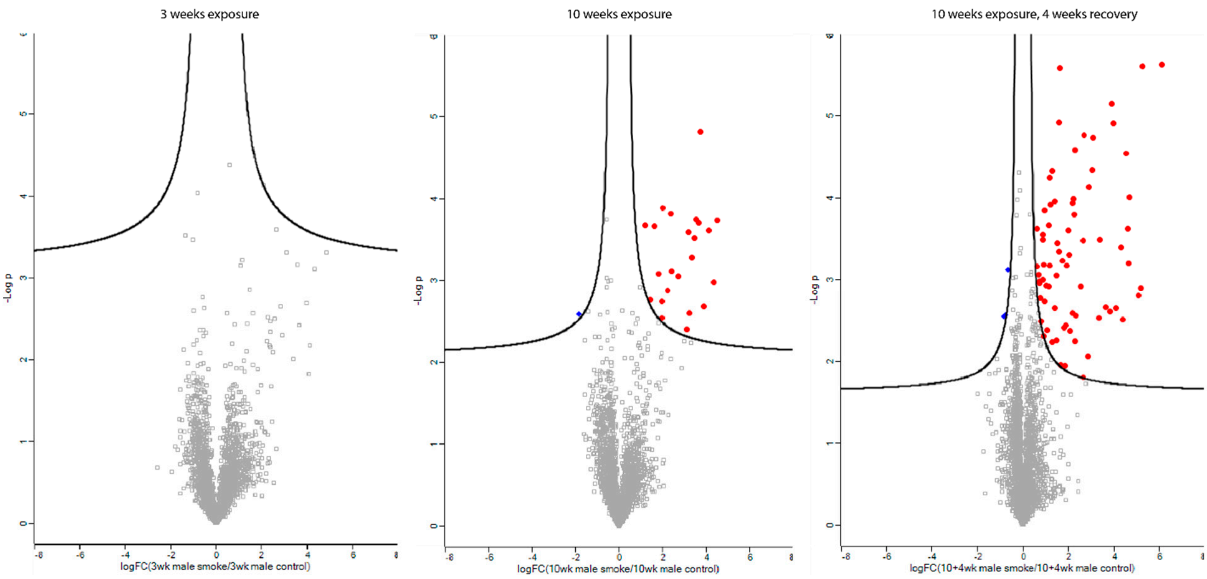

Figure S6. Validation of gene knockdown effect via qRT-PCR.

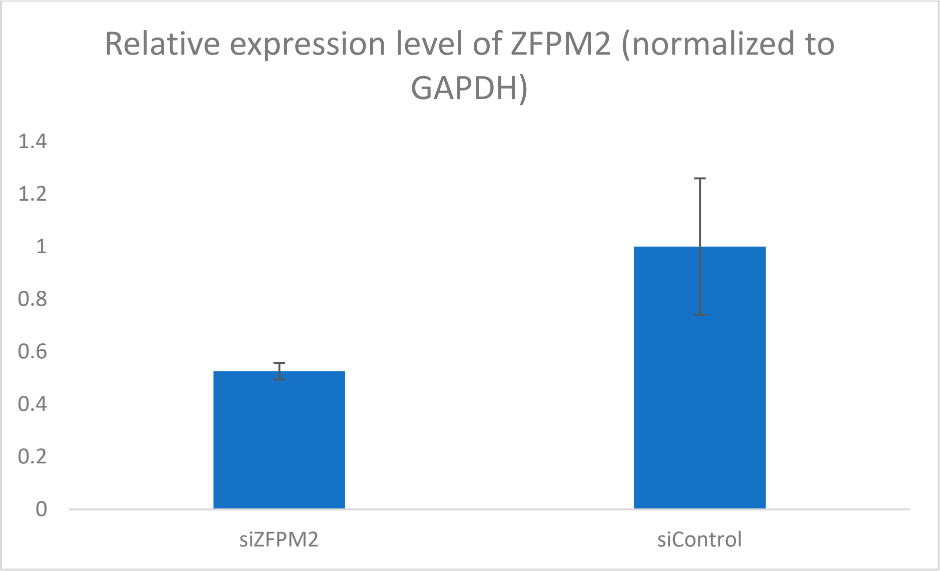

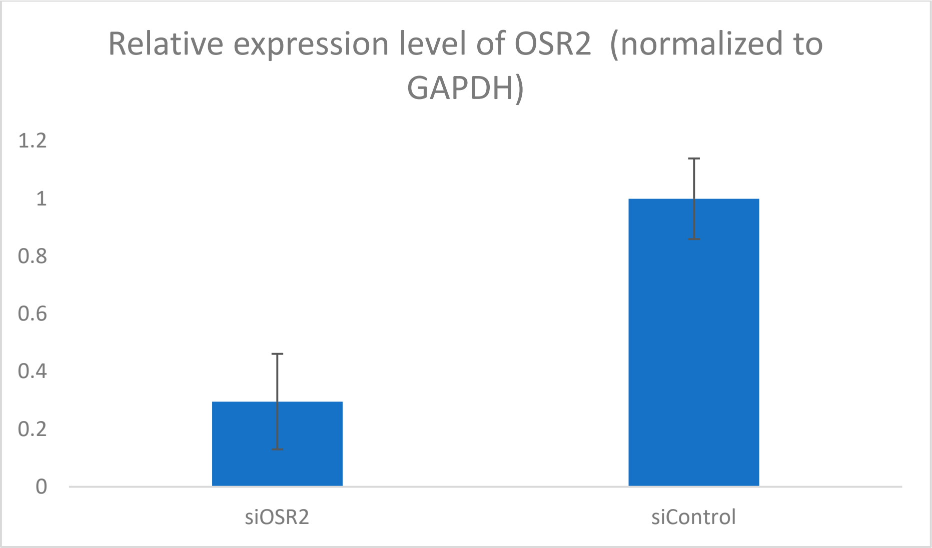

Figure S7. Cell proliferation assay of siRNA knockdowns of Psma6, Pdhx, Ruvbl1, Ywhaq

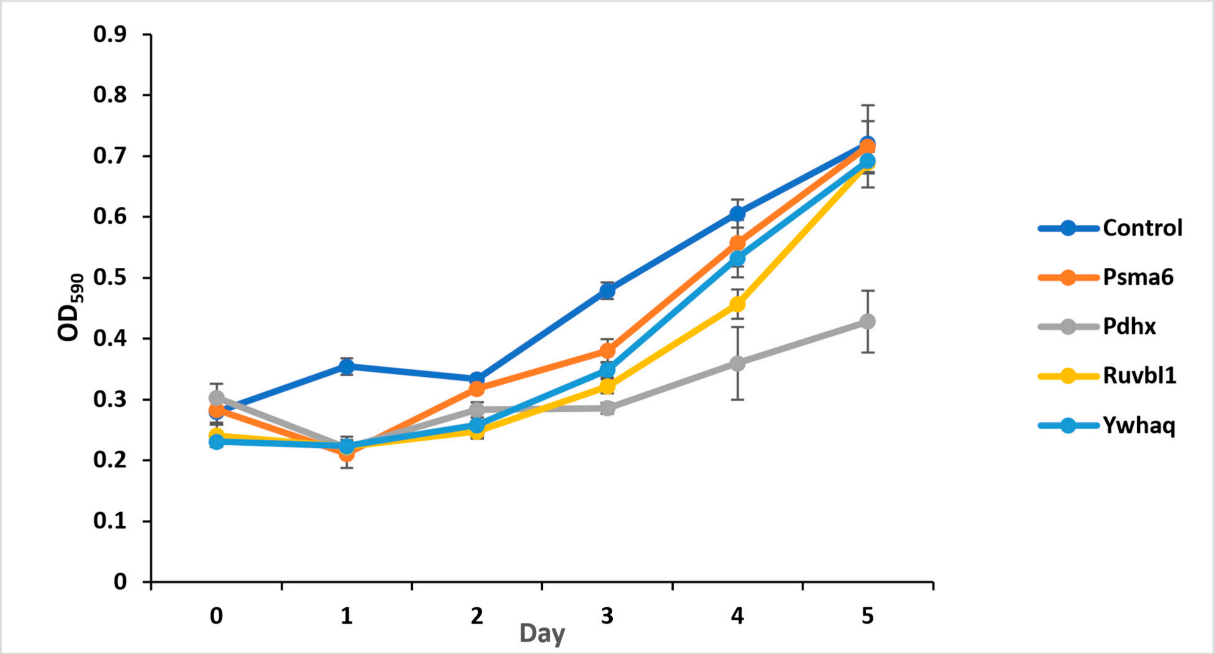

Supplement: Supplementary file 1 [file ijms-25-09365-s001.zip › ijms-3148586-supplementary.pdf]
